# Functional Screening Identifies MicroRNAs as Multi-Cellular Regulators of Heart Failure

**DOI:** 10.1038/s41598-019-41491-9

**Published:** 2019-04-15

**Authors:** Robin Verjans, Wouter J. A. Derks, Kerstin Korn, Birte Sönnichsen, Rick E. W. van Leeuwen, Blanche Schroen, Marc van Bilsen, Stephane Heymans

**Affiliations:** 1 Department of Cardiology, Cardiovascular Research Institute Maastricht (CARIM), Maastricht University, 6200 MD Maastricht, Limburg The Netherlands; 2 Former Cenix BioScience GmbH, 01307 Dresden, Saxony Germany; 3 Department of Physiology, Cardiovascular Research Institute Maastricht (CARIM), Maastricht University, 6200 MD Maastricht, Limburg The Netherlands; 4 Center for Molecular and Cardiovascular Biology, Department of Cardiovascular Sciences, 3001 Leuven, Vlaams-Brabant Belgium; 5 Netherlands Heart Institute, 3511 EP Utrecht, Utrecht The Netherlands

## Abstract

Heart failure (HF) is the leading cause of death in the Western world. Pathophysiological processes underlying HF development, including cardiac hypertrophy, fibrosis and inflammation, are controlled by specific microRNAs (miRNAs). Whereas most studies investigate miRNA function in one particular cardiac cell type, their multicellular function is poorly investigated. The present study probed 194 miRNAs –differentially expressed in cardiac inflammatory disease – for regulating cardiomyocyte size, cardiac fibroblasts collagen content, and macrophage polarization. Of the tested miRNAs, 13%, 26%, and 41% modulated cardiomyocyte size, fibroblast collagen production, and macrophage polarization, respectively. Seventeen miRNAs affected all three cellular processes, including miRNAs with established (miR-210) and unknown roles in cardiac pathophysiology (miR-145-3p). These miRNAs with a multi-cellular function commonly target various genes. In-depth analysis *in vitro* of previously unstudied miRNAs revealed that the observed phenotypical alterations concurred with changes in transcript and protein levels of hypertrophy-, fibrosis- and inflammation-related genes. MiR-145-3p and miR-891a-3p were identified to regulate the fibrotic response, whereas miR-223-3p, miR-486-3p, and miR-488-5p modulated macrophage activation and polarisation. In conclusion, miRNAs are multi-cellular regulators of different cellular processes underlying cardiac disease. We identified previously undescribed roles of miRNAs in hypertrophy, fibrosis, and inflammation, and attribute new cellular effects to various well-known miRNAs.

## Introduction

Heart failure (HF) is a huge burden on our society due to its high prevalence and mortality rates and is expected to form an even greater health problem in the future since effective treatment is still lacking^1,2^. The development of HF is characterized by hypertrophic growth of cardiomyocytes and excessive extracellular matrix deposition by proliferating and differentiated fibroblasts^3^. More recently it was recognized that HF is associated with a persistent pro-inflammatory state, characterized amongst others by infiltration of activated pro-inflammatory macrophages and polarization of these macrophages towards a pro-inflammatory (M1) phenotype^4,5^. Specific microRNAs (miRNAs) control these cellular processes driving HF development (reviewed in^6,7^). However, many miRNAs are solely studied for their involvement in only one cellular process, lacking insight into their multi-cellular function.

MiRNAs represent a class of small (±22 nucleotides in length) non-coding RNA molecules that negatively regulate target gene expression^8^. Specific miRNAs regulate the pathological processes underlying HF development^9–11^. MiRNA expression levels vary between cell types and change under pathological conditions^12^. Recent studies indicate that the effect of miRNAs is not restricted to the cell type(s) in which they are expressed, as they are secreted via extracellular vesicles. In this way, miRNAs act as paracrine signalling molecules on neighbouring cells^13–15^. Thus, despite the identification of various miRNAs associated with HF development, our understanding of their regulating potential and the cardiac cell types in which they exert their function remains incomplete.

Based on previous studies from our group, we selected 194 miRNAs that showed elevated or decreased expression levels primarily in hearts of HF patients of inflammatory cardiac diseases such as viral myocarditis and acute cardiac rejection^9,16^. Next, we developed a high-throughput phenotypic screening platform to assess their regulatory function in cardiomyocytes, fibroblasts and macrophages. MiRNA mimics were transfected into primary cultures of neonatal cardiomyocytes (nRCMs) and fibroblasts (nRCFs) to test their effect on the hypertrophic and fibrotic response, respectively. In addition, we screened these miRNAs for their influence on the polarization and activation of bone marrow-derived macrophages (BMDMs). The simultaneous testing of this subset of miRNAs in different cell types under unstimulated and stimulated conditions allowed us to assess the role of these miRNAs in the different cellular processes underlying HF development in an integrative manner and to unravel multi-cellular effects of multiple miRNAs. Seventeen miRNAs were identified to have a multi-cellular effect, affecting all three studied cellular processes in parallel. These 17 miRNAs share common target genes in the human failing heart, of which 15 genes are targeted by at least 7 out of the 17 selected miRNAs. We confirmed targeting of these genes by overexpressing miR-145-3p in cardiomyocytes, fibroblasts, and macrophages, decreasing transcript expression of various genes in multiple cell types in parallel. These findings mark these genes and their miRNA-controlled expression as a contributing factor to human heart failure progression.

Thirteen miRNAs were selected for further in-depth analysis to elucidate their hypertrophy-, fibrosis- and inflammation-regulating properties at transcript and protein level. Selected miRNAs have never before been studied for their regulatory function in cardiac pathophysiology and were identified in at least one of the three screens as a pronounced process effector. Combining miRNA-induced phenotypical changes with modulation of corresponding marker expression led to the identification of fibrosis- (miR-145-3p and miR-891a-3p) and inflammatory-regulating miRNAs (miR-223-3p, miR-486-3p, and miR-488-5p). These findings identify previously undescribed roles of miRNAs in different cellular processes underlying cardiac disease and attribute new cellular effects to various well-known miRNAs, confirming that miRNAs function as multi-cellular regulators.

## Results

**Figure 1:**
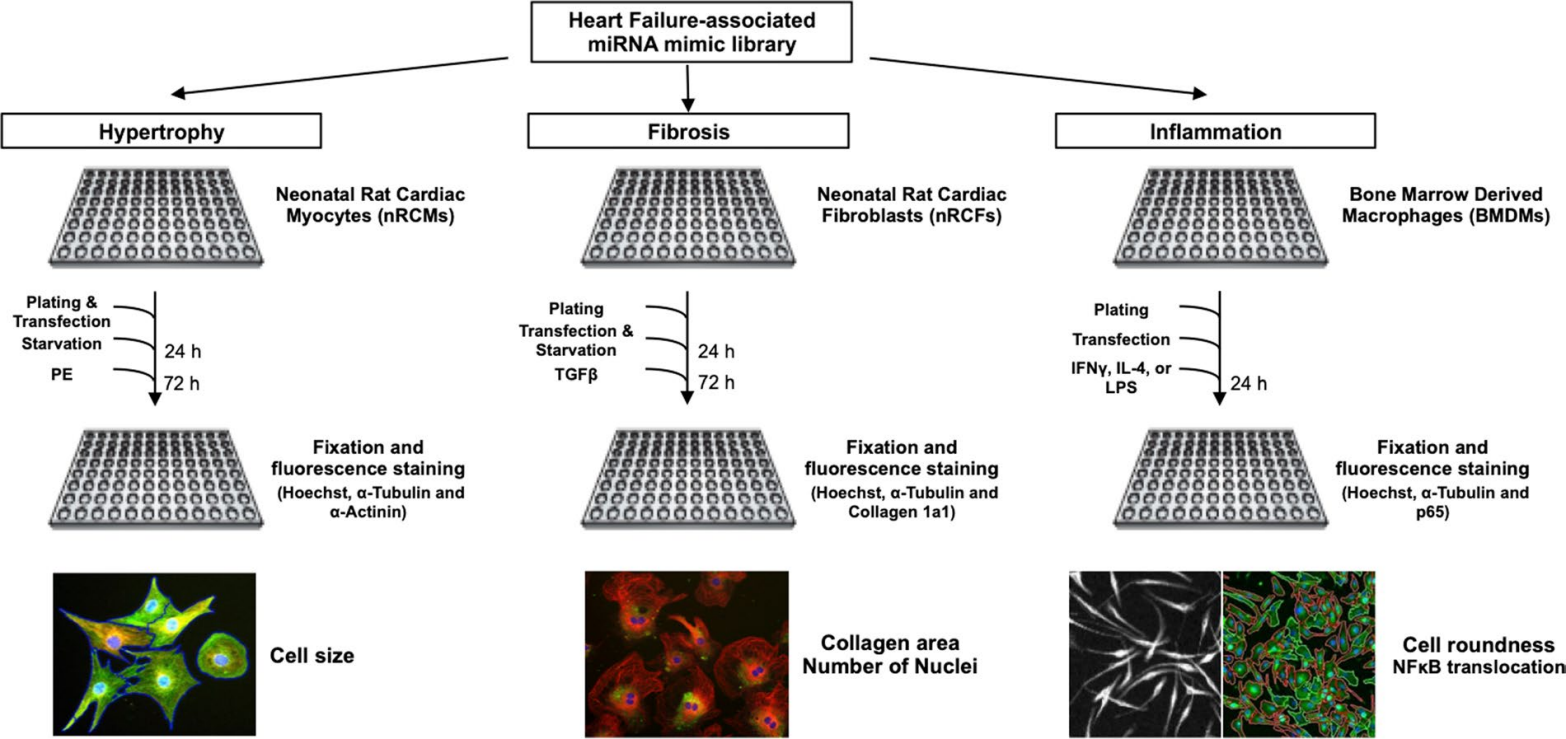
Phenotypical screening using a HF-associated miRNA library was performed in parallel for multiple HF-underlying processes. Effects induced under control and HF-mimicking conditions by miRNA mimics on hypertrophy, fibrosis and inflammation were studied using nRCMs, nRCFs, and BMDMs. Using automated image analysis, quantified read-outs were cardiomyocyte size, cardiac fibroblast number and collagen area, and macrophage roundness and NFκB nuclear translocation.

We assembled a library of 194 miRNA mimics based on their differential cardiac expression in patients diagnosed with viral myocarditis, patients undergoing cardiac transplantation, largely overlapping with animal models of similar inflammatory cardiac disease (Supplementary Table S1). To screen for involvement of these miRNAs in cellular processes driving heart failure (HF), this HF-associated miRNA mimic library was introduced in three primary cell types held accountable for pathological cardiac remodelling (Fig. 1).

### MiRNAs affect cardiomyocyte hypertrophy

**Figure 2:**
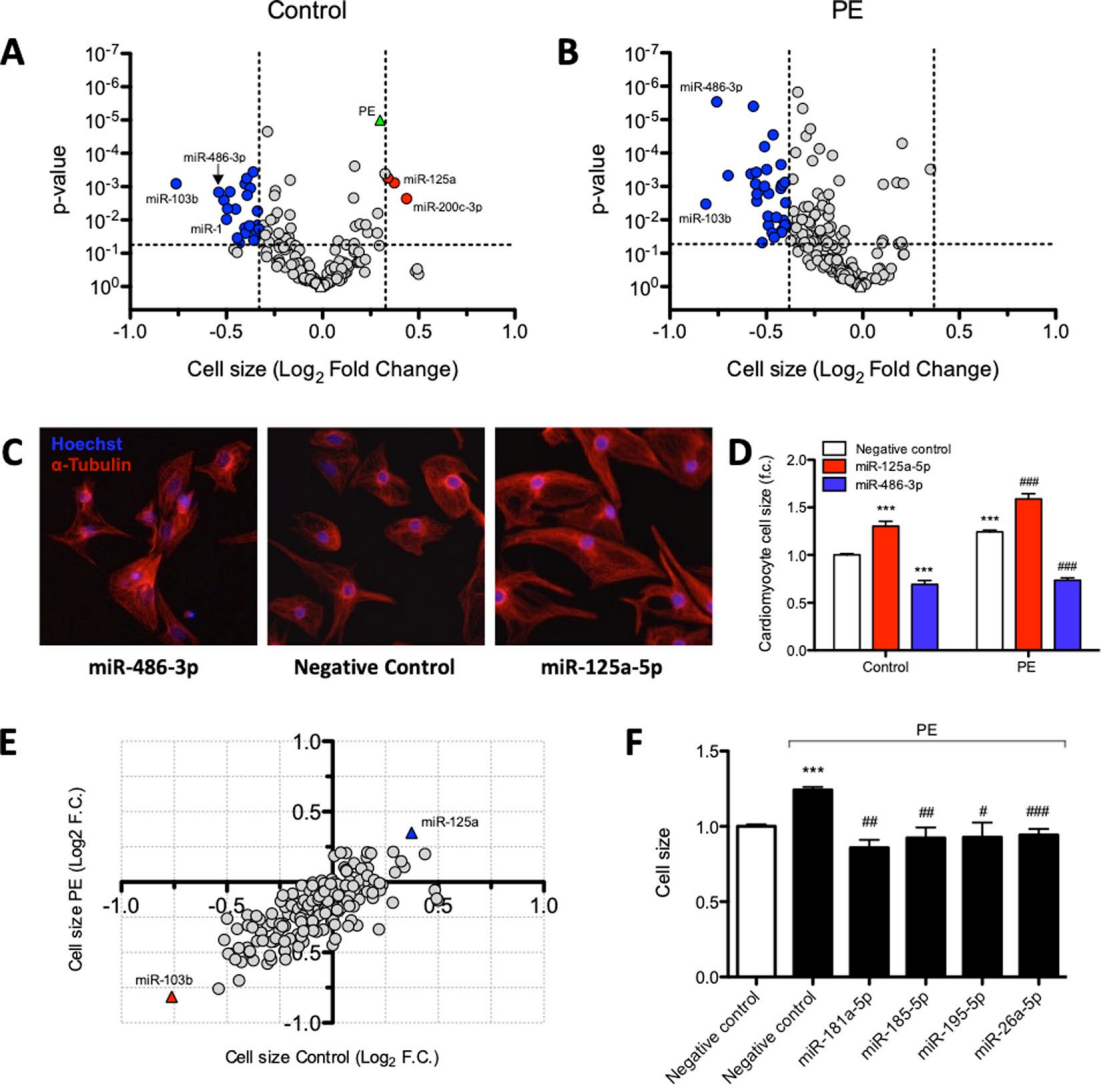
Hypertrophy screen identifies HF-associated miRNAs that affect cardiomyocyte cell size. MiRNA mimic-induced changes in cardiomyocyte cell size without (panel A) or with (panel B) PE stimulation. Values represent cardiomyocyte cell size expressed as log_2_ fold change, normalised to negative control mimic-transfected cells within the same condition, n = 6 replicates per miRNA mimic per condition. MiRNA mimics that induce a significant (Unpaired t-test, P < 0.05) effect on cell size, deviating more than 2x STDEV from negative control (indicated by vertical lines) are highlighted in red or blue. The PE-induced increase in cell size is represented in green in panel A. (**C**) Representative images of the miRNAs with pronounced effects on cells size, miR-486-3p and miR-125a-5p, under unstimulated conditions. (**D**) Quantification of cardiomyocyte cell size under control and PE-stimulated conditions after transfection of miR-486-3p and miR-125a-5p mimics (Unpaired t-test, P < 0.001). (**E**) The majority of miRNA mimics affect cardiomyocyte cell size irrespectively of stimulation. (**F**) Several miRNA mimics blunted PE-induced increase in cell size (Unpaired t-test, P < 0.05); presented values are expressed as means ± standard error, ***denotes P < 0.001 versus unstimulated negative control, ^#^denotes P < 0.05, ^##^denotes P < 0.01, ^###^denotes P < 0.01 versus PE-stimulated negative control.

MiRNA mimics were transfected into unstimulated and PE-stimulated neonatal rat cardiomyocytes (nRCMs) and cell size was quantified to determine their involvement in hypertrophic growth. Of the 194 miRNA mimics that were introduced into nRCMs, 25 affected nRCM size in unstimulated conditions according to a pre-defined threshold (Fig. 2A). Three miRNAs, miRNA-200c-3p, miR-125a-5p, and miR-92b-3p, significantly increased cell size relative to negative control-mimic transfected cells (further referred to as negative control), and 22 miRNAs were found to reduce cell size (Fig. 2A). Among the latter, miR-486-3p, miR-145-3p, and miR-1 reduced cell size significantly.

PE stimulation triggered the hypertrophic response in nRCMs, characterized by 0.30 log_2_ fold increase in cell size (Fig. 2A) and increased transcript levels of hypertrophic markers αSKA, ANF and BNP (Supplementary Fig. S1A–C). Although not fulfilling our criteria, 8 miRNAs induced an additional increase in cell size compared to negative control under PE-stimulated conditions (Fig. 2B). In contrast, 31 miRNAs decreased cardiomyocyte cell size under PE-stimulated conditions, among which miR-103b, miR-151a-3p, miR-145-3p, miR-486-3p, and miR-891a-3p (Fig. 2B). Transfection with 13 miRNA mimics (including miR-148a-3p, miR-145-3p, miR-486-3p, and miR-891a-3p) significantly decreased cell size under both control and PE-stimulated conditions (Supplementary Table S1).

Although most miRNA-induced alterations in cardiomyocyte size were independent of stimulation conditions (Fig. 2C–E), transfection with 13 miRNA mimics, including miR-181a-5p, miR-185-5p, miR-195-5p, and miR-26a-5p (Fig. 2F), decreased cardiomyocyte cell size only under PE-treated conditions while having no significant effect under unstimulated conditions (Supplementary Table S2).

Table 1 highlights the top miRNAs identified to increase or decrease cardiomyocyte cell size under control conditions and upon PE stimulation. Interestingly, miR-125a-5p and miR-103b have the most pronounced effect on cell size independent of PE stimulation. These results indicate that a large fraction of the selected HF-associated miRNAs is able to regulate cardiomyocyte hypertrophy.

**Table 1:** Top miRNAs able to increase or decrease cardiomyocyte cell size.

miRNA	Control		PE-stimulated	
	Cell size (Log_2_ f.c.)	p-value	Cell size (Log_2_ f.c.)	p-value
miR-200c-3p	0.437	0.002	0.200	0.047
miR-125a-5p	0.374	0.001	0.351	0.000
miR-92b-3p	0.339	0.001	0.108	0.441
miR-103b	−0.761	0.001	−0.814	0.003
miR-486-3p	−0.540	0.001	−0.757	0.000
miR-103a-3p	−0.513	0.003	−0.411	0.010
miR-1	−0.500	0.010	−0.260	0.058
miR-214-3p	−0.494	0.005	−0.509	0.001

### MiRNAs affect cardiac fibroblast collagen area and proliferation

**Figure 3:**
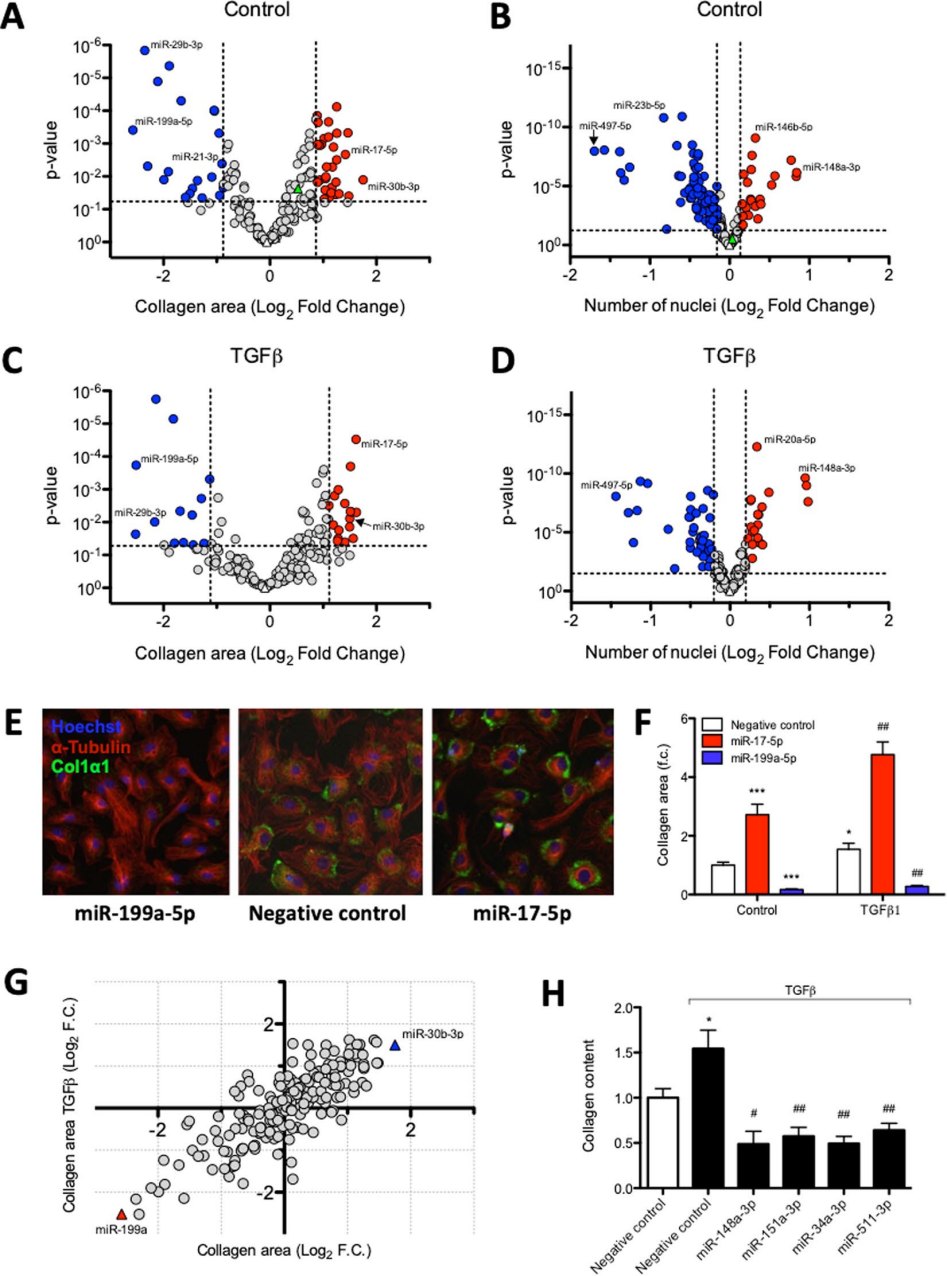
Effect of miRNA mimics on cardiac fibroblast proliferation and collagen production upon transfection. Vulcano plots showing collagen area and number of nuclei for individual miRNA mimics without (panel A and B) or with (panel C and D) TGFβ stimulation. Values represent log_2_ fold change, normalised to negative control mimic-transfected cells within the same condition, n = 6 replicates per miRNA mimic per condition. MiRNA mimics that significantly (Unpaired t-test, P < 0.05) increased or reduced these readouts according to previously described criteria are highlighted in red or blue, respectively. As a point of reference, the effects that are reached by TGFβ stimulation of untransfected controls are represented as green triangles in both (panels A and B). (**E**) Representative images of miR-199a and miR-17-5p. (**F**) Quantification of cardiac fibroblast collagen area under control and TGFβ-stimulated conditions upon transfection of miR-199a and miR-17-5p mimics (Unpaired t-test, P < 0.05). (**G**) MiRNA-mimic induced changes in collagen area in the presence and absence of treatment. (**H**) Different miRNA mimics decreased collagen area only upon TGFβ stimulation (Unpaired t-test); presented values are expressed as means ± standard error, *denotes P < 0.05, ***denotes P < 0.001 versus unstimulated negative control, ^#^denotes P < 0.05, ^##^denotes P < 0.01 versus TGFβ-stimulated negative control.

To determine the effect of the HF-associated miRNAs on the fibrotic response, we quantified collagen production and proliferation of neonatal rat cardiac fibroblasts (nRCFs) upon miRNA mimic introduction. Figure 3A illustrates the log_2_ fold change in collagen area for each mimic relative to negative control under unstimulated conditions. A large number of miRNA mimics (30) significantly increased collagen area, including miR-379-3p, miR-151a-5p, and miR-30a-5p. Twenty miRNA mimics decreased collagen area, among them are miR-199a-5p, miR-21-3p, miR-103a-2-5p, and several members of the miR-29 family which are known to target several collagen subtypes^11^, including COL1*α*1. A large fraction of the tested miRNA mimics was able to significantly increase (22) or decrease (95) the number of cardiac fibroblasts, as reflected by the counted nuclei (Fig. 3B). Interestingly, 34 miRNA mimics altered both cardiac fibroblast collagen content and number of nuclei under baseline conditions (Supplementary Table S1). Again, among these are members of the miR-29 family known for their anti-fibrotic effects^11,17^.

To initiate a pro-fibrotic response, cardiac fibroblasts were stimulated with TGFβ, resulting in an upregulation of the fibrosis marker connective tissue growth factor (CTGF)(Supplementary Fig. S2A) and an increase in COL1*α*1 positive stained cell area (further referred to as collagen area) (Fig. 3A). Under TGFβ-stimulated conditions, our screen revealed 17 and 13 miRNAs to increase or decrease collagen area, respectively (Fig. 3C). In the presence of TGFβ, 20 miRNA mimics increased and 45 decreased the number of nuclei (Fig. 3D).

MiR-199a and miR-30b-3p have the most outspoken effect on collagen area, hardly affected by the TGFβ stimulus (Fig. 3E–G). Strikingly, transfection of mimics for miR-148a-3p, miR-151a-3p, miR-34a-3p, and miR-511-3p did not affect fibroblast collagen area under unstimulated conditions but decreased collagen area only in combination with TGFβ treatment, thereby blunting the fibrotic response (Fig. 3H).

Table 2 lists the top miRNAs with the most pronounced effects on cardiac fibroblast collagen content in both directions under control and TGFβ-stimulated conditions. A large fraction of the selected miRNAs is able to affect collagen production and proliferation of cardiac fibroblasts.

**Table 2:** Top miRNAs able to increase or decrease collagen content.

miRNA	Control		TGFβ-stimulated	
	Collagen area (Log_2_ f.c.)	p-value	Collagen area (Log_2_ f.c.)	p-value
miR-30b-3p	1.752	0.013	1.108	0.003
let-7e-5p	1.481	0.040	1.072	0.143
miR-7-5p	1.465	0.000	1.073	0.025
miR-17-5p	1.420	0.002	1.617	0.000
miR-152-5p	1.299	0.034	0.530	0.265
hsa-miR-199a-5p	−2.575	0.000	−2.515	0.000
hsa-miR-29b-3p	−2.350	0.000	−2.164	0.010
hsa-miR-103a-2-5p	−2.297	0.000	−2.524	0.023
hsa-miR-29c-3p	−2.109	0.000	−1.896	0.082
hsa-miR-29a-3p	−1.991	0.013	−1.988	0.050

### Macrophage polarization and activation is affected by miRNAs

**Figure 4:**
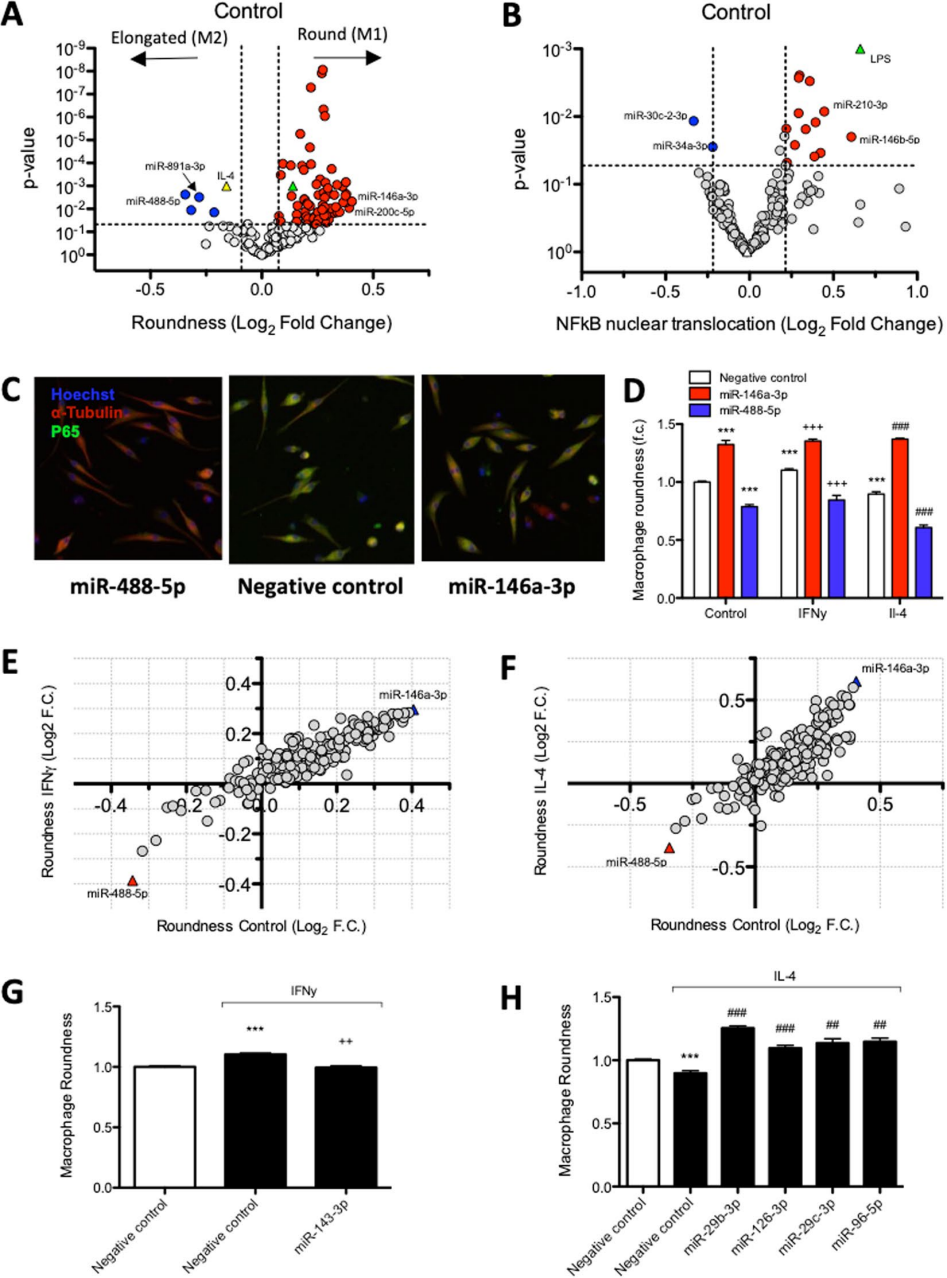
Inflammation screen identifies HF-associated miRNAs affecting macrophage roundness and NFκB nuclear translocation. MiRNA mimic-induced changes in macrophage roundness (**A**) and NFκB nuclear translocation (**B**) under control conditions. Values are represented as log_2_ fold change, normalised to negative control mimic-transfected cells within the same condition, n = 6 replicates per miRNA mimic per condition. MiRNA mimics that significantly (Unpaired t-test, P < 0.05) increased or reduced these readouts are highlighted in red or blue. In panel A, polarization effects on cell roundness in negative control mimic-transfected cells induced by IFNγ and IL-4 stimulation are represented as yellow and green triangles. In panel B, LPS-induced effect on NFκB nuclear translocation in negative control mimic-transfected cells is represented as a green triangle. (**C**) Representative images of miR-488-5p and miR-146a-3p with pronounced effects on macrophage roundness and NFκB nuclear translocation. (**D**) Quantification of macrophage roundness under control, INFγ, and IL-4-stimulated conditions upon transfection with miR-488-5p and miR-146a-3p mimics (Unpaired t-test, P < 0.001). The majority of miRNA mimics affect macrophage roundness to a comparable extent under control, IFNγ (**E**) or IL-4 (**F**) stimulated conditions. (**G**) MiR-143-3p prevents IFNγ-induced increase in roundness, while having no effect under basal conditions (Unpaired t-test, P < 0.01). (**H**) Different miRNA mimics increase roundness only in combination with IL-4 stimulation (Unpaired t-test, P < 0.01), presented values are expressed as means ± standard error, ***denotes P < 0.001 versus unstimulated negative control, ^++^denotes P < 0.01 and ^+++^denotes P < 0.001 versus IFNγ-stimulated negative control, ^##^denotes P < 0.01 and ^###^denotes P < 0.001 versus IL-4-stimulated negative control.

To complete our evaluation of the impact of HF-associated miRNAs on HF pathways, we evaluated the effect of the miRNA mimics on macrophage polarization and activation. Macrophages reflect the most abundant immune cell type of the heart^18^ and depending on their polarization status, macrophages exhibit differences in morphology, cytoskeletal organization, functional characteristics, and migrating capacity^19^. The effect of individual mimics on macrophage polarization was quantified under unstimulated (M0), IFNγ (M1) and IL-4 (M2) stimulated conditions. Morphological appearance of macrophages is a marker of polarization status in BMDMs, with M1 (pro-inflammatory) macrophages showing a round and M2 (anti-inflammatory) polarized macrophages having an elongated phenotype^20^. In agreement, BMDM M1 and M2 polarization resulted in a 0.14 and −0.16 log_2_ fold change in round classified cells (Fig. 4A). Phenotypical alterations upon inflammatory stimulus treatment are accompanied by differential expression of molecular markers representing functional changes in coherence with M1 or M2 polarization (Supplementary Fig. S3A–C).

In unstimulated macrophages, four miRNA mimics decreased macrophage roundness: miR-488-5p, miR-34a-3p, miR-891a-3p, and miR-128-3p. A large number (76) of miRNAs significantly increased roundness of BMDMs (Fig. 4A), among them miR-200c-5p, miR-148b-3p, and miR-155-5p, the latter being a known inducer of M1 polarization^21^. Comparable results were found for macrophages polarised towards M1 (IFNγ) and M2 (IL-4) (Supplementary Fig. S3D,E). MiR-155, a well-known pro-inflammatory miRNA^9,22^, caused a pronounced and significant increase in roundness in all conditions (Supplementary Table S1).

MiR-146a-3p and miR-488-5p have the most pronounced effect on macrophage roundness under basal conditions (M0), as well as IFNγ (M1) and IL-4 (M2) treated conditions (Fig. 4E,F). MiR-143-3p diminished the IFNγ-induced increase in macrophage roundness without altering macrophage morphology under unstimulated conditions, indicative of a M1 polarization-inhibiting function (Fig. 4G). Similarly, a total of 16 miRNAs, including miR-126-3p, miR-29b-3p, miR-29c-3p, and miR-96-5p, increased macrophage roundness only upon IL-4 stimulation (Fig. 4H) (Supplementary Table S3), suggesting that these miRNAs comprise the ability to inhibit M2-polarization. The top miRNAs able to promote or decrease macrophage roundness, as identified under control conditions, along with their effect on NFκB nuclear translocation, are shown in Table 3.

**Table 3:** Top miRNAs able to increase or decrease macrophage roundness.

miRNA	Control	
	Roundness (Log_2_ f.c.)	p-value
miR-146a-3p	0.404	0.005
miR-200c-5p	0.392	0.009
miR-30a-3p	0.380	0.002
miR-148b-3p	0.378	0.005
miR-218-5p	0.378	0.001
miR-488-5p	−0.343	0.002
miR-34a-3p	−0.317	0.011
miR-891a-3p	−0.281	0.003
miR-128-3p	−0.214	0.014

The effect of individual mimics on the activation of BMDMs was quantified using an NFκB nuclear translocation assay either stimulated with or in absence of LPS. Activation of negative control mimic-transfected BMDMs with LPS resulted in a significant 0.66 log_2_ fold increase in NFκB nuclear translocation (Fig. 4B). In the absence of LPS stimulation, only miR-30c-2-3p and miR-34a-3p were identified to significantly decrease NFκB nuclear translocation. Thirteen miRNAs were found to significantly increase NFκB nuclear translocation, including miR-210-3p, miR-590-5p, miR-96-5p, and miR-152-3p. MiR-146b-5p, a positive regulator of the NFκB pathway^23^, induced the strongest increase in NFκB nuclear translocation. Supplementary Fig. S3F shows the log_2_ fold change in NFκB nuclear translocation for each individual miRNA mimic in macrophages activated with LPS. Although the relationship is only moderate, phenotypical alterations in macrophage shape and NFκB nuclear translocation upon miRNA mimic transfection correlate significantly (p < 0.001; R^2^ = 0.374), supporting that differences in macrophage roundness are accompanied with functional alterations (Supplementary Fig. S3G).

### MiRNAs with multi-cellular effects target common genes

**Figure 5:**
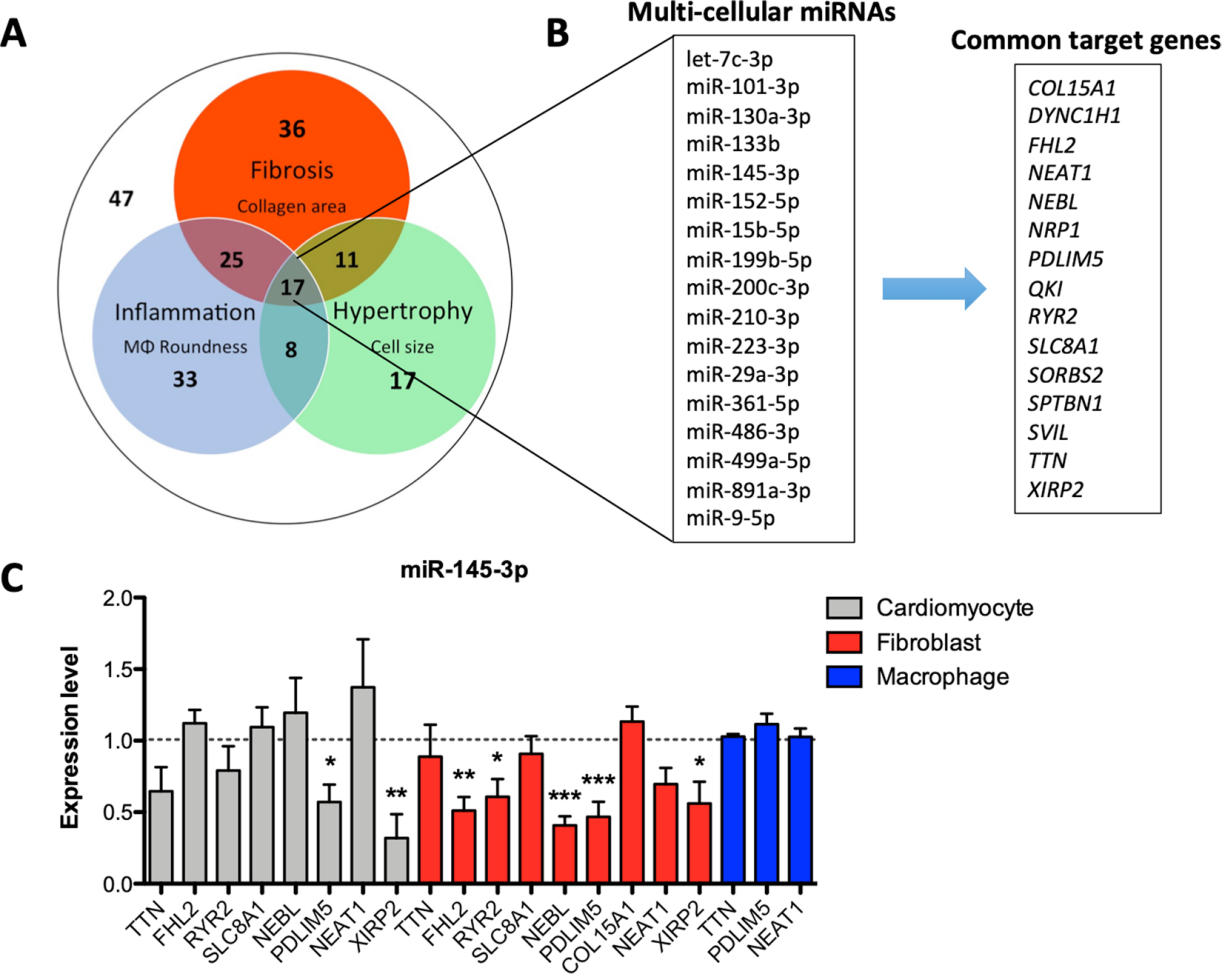
MiRNAs with a multi-cellular function control the expression of common target genes. (**A**) The majority of the miRNAs mimics affect at least one pathological process. Included read outs are cell size, collagen content, and cell roundness, for the hypertrophy, fibrosis, and inflammation screen, respectively. (**B**) The 17 miRNAs with a multi-cellular function target various common genes. (**C**) Overexpression of multi-cellular miR-145-3p decreases the transcript expression of various target genes in multiple cell types in parallel.

The vast majority of miRNAs (76%) affected at least one of the HF-related readouts without any additional stimulus (Fig. 5A). Of the total miRNA-library, 27% of the selected miRNAs significantly altered cardiomyocyte size, almost half (46%) of the tested miRNA mimics were able to significantly affect cardiac fibroblast collagen area, and 43% was identified as modulators of macrophage roundness. The overlap between the different processes is substantial, marking the relatively high number of miRNAs (32%) affecting more than one process. Notably, 17 miRNAs influenced each of the HF-underlying processes to a significant extent (Fig. 5B and Supplementary Table S4).

To study the mechanism underlying the regulatory function of these 17 miRNAs, we identified common target genes by using the publically available data deriving from the study of Spengler *et al*.^24^. This study used AGO2 crosslinking immunoprecipitation coupled with high throughput sequencing (HITS-CLIP) of bound RNA interaction sites, resulting in a complete description of the transcriptome-wide map of miRNA targeting events in the human failing heart. We identified 15 genes which expression is under the control of at least 7 out of the 17 selected miRNAs in the human failing heart (see the Materials and Methods section for a more detailed description of the identification of target genes), including Four And A Half LIM Domains 2 (FHL2), Ryanodine Receptor 2 (RYR2), Nebulette (NEBL), PDZ And LIM Domain 5 (PDLIM5), and Xin Actin Binding Repeat Containing 2 (XIRP2) amongst others (Fig. 5B). We validated targeting of the common genes upon overexpression of miR-145-3p in cardiomyocytes, fibroblasts, and macrophages (Fig. 5C). Although miR-145-3p did not have a significant effect on the expression of target genes in macrophages, miRNA overexpression decreased the levels of FHL2, NEBL, and RYR2 in cardiac fibroblasts significantly. More importantly, overexpression of miR-145-3p decreased the transcript levels of PDLIM5 and XIRP2 in both cardiomyocytes and fibroblasts, marking these genes as important players in the regulatory function of miR-145-3p in both cardiomyocytes and fibroblasts. These results confirm that multi-cellular miRNAs have multiple target genes in common and that these miRNAs exert their function, at least partly, through inhibition of the same target genes in various cell types.

### MiRNAs modulate HF-driving processes at transcriptional and protein level

Thirteen miRNAs were selected for further in-depth investigation to study their regulatory function of cellular processes driving HF at transcript and protein level. These miRNAs have not been functionally assessed in the context of heart failure before, and were selected based on their ability to strongly modulate at least one HF-underlying process in our screening approach. Supplementary Table S5 shows the selected miRNAs and their induced phenotypical changes upon transfection in the relevant primary cell type.

**Figure 6:**
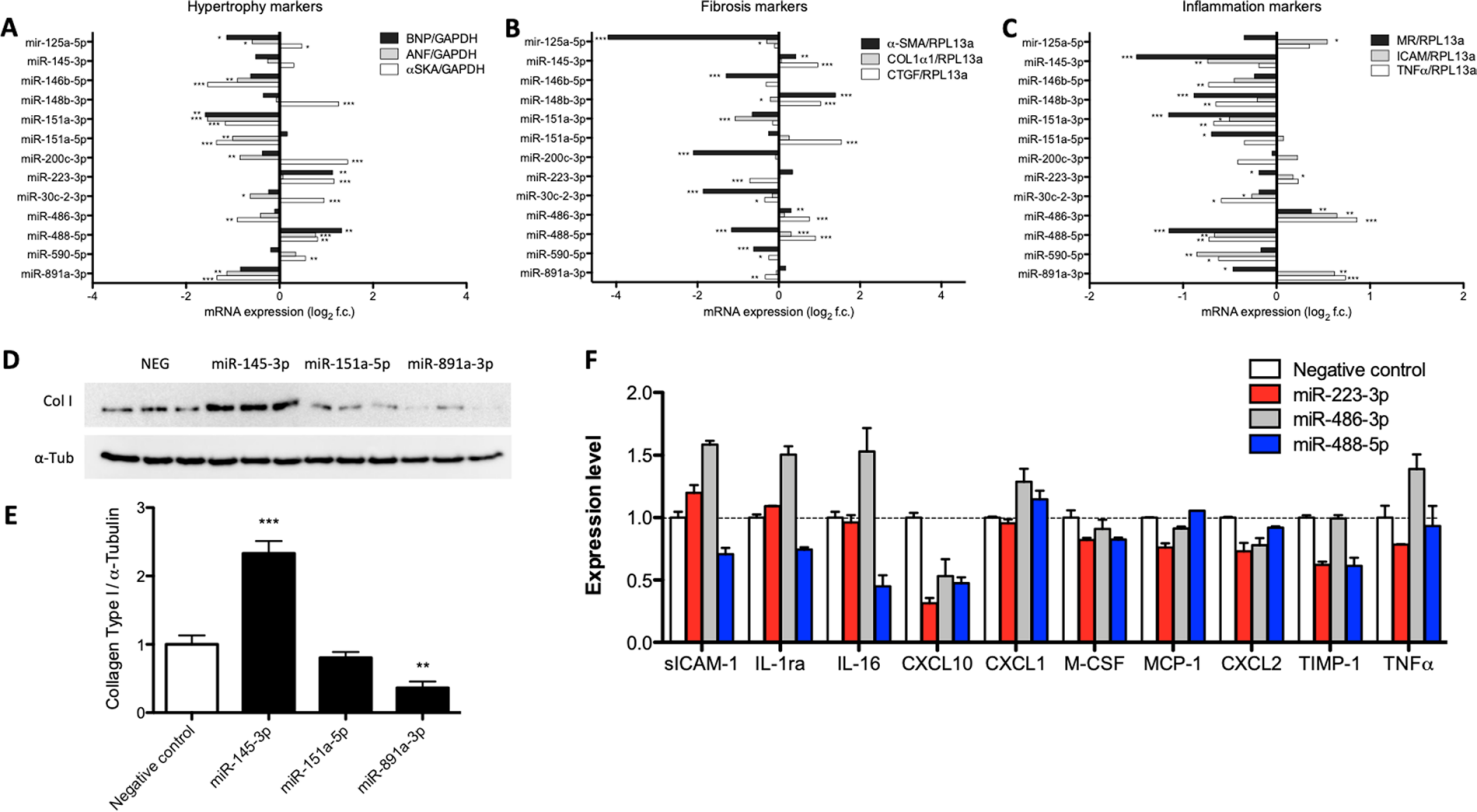
MiR-145-3p and miR-891a-3p control the fibrotic response while miR-223-3p, miR-486-3p, and miR-488-5p control the inflammatory response. Quantification of mRNA levels of (**A**) hypertrophic (ANF, BNP, and αSKA), (**B**) fibrotic (CTGF, COL1α1, and α-SMA), and (**C**) inflammatory markers (TNFα, ICAM and MR) in respectively nRCMs, nRCFs, or BMDMs after miRNA mimic transfection (Unpaired t-test). (**D**) Representative western blots of collagen type I (Col I) and normalizer α-Tubulin (α-Tub) performed with cell lysates of nRCFs transfected with negative control (NEG), miR-145-3p, miR-151a-5p, and miR-891a-3p mimics. Samples were run on the same gel and blot, different regions of the blot were cropped to quantify Col I and a-Tub, and every sample per protein was acquired under the same exposure conditions. (**E**) Western blot quantification reveals increased and decreased Collagen Type I protein expression in miR-145-3p and miR-891a-3p transfected nRCFs, respectively (One Way ANOVA). (**F**) Levels of cytokines and chemokines determined using a Mouse Cytokine Array in the supernatant of BMDMs transfected with miR-223-3p, miR-486-3p, or miR-488-5p mimics. In (**A**–**C**), bar graphs display mean log_2_ fold change in comparison with negative control transfected cells ± standard error. In (**E**,**F**), bar graphs display mean fold change in comparison with negative control transfected cells ± standard error. *Denotes P < 0.05, **denotes P < 0.01, and ***denotes P < 0.001 versus unstimulated negative control.

To determine if selected miRNAs regulate the hypertrophic response in cardiomyocytes at transcript level, we quantified the hypertrophic markers alpha-skeletal actin (αSKA), atrial natriuretic factor (ANF), and brain natriuretic peptide (BNP) (Fig. 6A). Transfection of miRNA mimics for miR-488-5p induced expression of all three hypertrophic markers, suggesting a pro-hypertrophic response at transcript level. On the contrary, ANF, BNP and αSKA mRNA levels decreased significantly after overexpression of miR-151a-3p, suggesting an anti-hypertrophic effect.

Regulation of the fibrotic response at transcript level was determined by quantification of expression levels of myofibroblast marker alpha-smooth muscle actin (α-SMA), as well as collagen markers connective tissue growth factor (CTGF) and collagen 1a1 (Col1α1) (Fig. 6B). Both miR-151a-5p and miR-145-3p were identified to increase cardiac fibroblast collagen area and accordingly induced transcript expression of collagen marker CTGF or both α-SMA and CTGF, respectively. On the other hand, miR-891a-3p strongly decreased collagen area and CTGF expression upon transfection. To further determine the fibrotic response-regulatory function of these miRNAs, we determined their effect on protein expression of Collagen type 1 (Fig. 6D,E). Although transfection of miR-151a-5p mimics did not have any effect, forced expression of miR-145-3p increased protein expression of collagen type 1 dramatically, indicating a pro-fibrotic role of miR-145-3p in cardiac fibroblasts. Conversely, transfection of miR-891a-3p mimics decreased Collagen type 1 protein expression in cardiac fibroblasts, implying an anti-fibrotic function in cardiac fibroblasts.

In-depth characterisation of inflammation-modulating properties was done by quantification of anti-inflammatory marker mannose receptor (MR), as well as pro-inflammatory markers tumor necrosis factor-alpha (TNFα) and intercellular adhesion molecule (ICAM) mRNA expression levels (Fig. 6C). Transfection of miR-223-3p and miR-486-3p strongly increased macrophage roundness and induced transcript expression of TNFα and all three inflammation markers, respectively. On the other hand miR-488-5p decreased the expression of all three inflammatory markers, in line with the observed decreased macrophage roundness. Further investigation of these immune-modulating miRNAs was done via characterisation of the secreted cytokine and chemokine profile (Fig. 6F). MiR-223 mimics altered the levels of different cytokines in the supernatant of cultured macrophages, increasing the levels of the pro-inflammatory cytokine soluble (s)ICAM-1, but decreasing TNFα levels. Notably, miR-486-3p induced a strong upregulation of various pro-inflammatory cytokines/chemokines, including sICAM-1, IL-1ra, IL-16, CXCL1, and TNFα. These results suggest that miR-223-3p and miR-486-3p alter macrophage polarisation and activation, promoting the inflammatory response. On the other hand, macrophages transfected with miR-488-5p mimics showed decreased levels of almost all detectable cytokines, implying an anti-inflammatory function.

## Discussion

In the present study we employed high-throughput phenotypical screening in three different primary cell types to explore the effect of 194 cardiac inflammation-related miRNAs on three pivotal processes underlying HF development, namely cardiomyocyte hypertrophy, fibrosis, and macrophage polarization. Novel cellular functions of well-studied miRNAs and less well-known miRNAs putatively involved in HF progression were identified. This comprehensive screening approach unveils the complimentary cellular actions of various miRNAs in different cell types involved in cardiovascular disease and opens new avenues for the treatment of HF via miRNA-directed therapeutic approaches. Previous screens of miRNAs on cardiac phenotypes focussed on a single process, either on myocyte hypertrophy^25^ or proliferation^26^, and did not include an additional stimulus to mimic pathological processes^27^. By simultaneously screening in three primary cell types under basal conditions as well as after applying a pathogenic stimulus, the current approach allows a better understanding of the multi-cellular role of these miRNAs.

Using our phenotypical screening approach, miRNAs not known for their role in cardiomyocyte hypertrophy were identified, besides confirming known anti- and pro-hypertrophic effects of others, such as miR-1^10^ and miR-19a-3p^28^. Interestingly, miR-125a-5p, miR-200c-3p, and miR-92b-3p mimics are examples of miRNAs of which a novel pro-hypertrophic function was discovered. These miRNAs had previously only been associated to cardiac diseases, based on their altered expression in cardiac tissue biopsies (miR-125a-5p^29^ and miR-200c-3p^30^), or in the blood (miR-92b^31^). Many miRNAs reduced cardiomyocyte cell size, and here the unknown miR-486-3p had the most outspoken anti-hypertrophic effect. While our “unstimulated” cardiomyocyte screen confirms and extends on identified miRNAs in a previously performed screen^25^, we additionally found that several miRNAs have a unique PE-dependent effect. Transfection of 13 miRNA mimics, including miR-185-5p, prevented the PE-induced hypertrophic response, suggesting miRNA-mediated targeting of genes specifically involved in alpha-adrenergic downstream signalling. Of note, cardiac miR-185 expression was shown earlier to be down-regulated during cardiac hypertrophy^32^ and to play an important anti-hypertrophic role via targeting multiple genes involved in the hypertrophic process, including Ncx1, Nfatc3, CAMK2d^32^, RhoA, and Cdc42^33^.

Investigating the fibrosis-modulating potential of our HF-associated miRNA library identified unknown miRNAs with anti- or pro-fibrotic properties and confirmed the effect of others, such as the well-described members of the miR-29 family, known to target collagen production^11^, and miR-24-1-5p, known to reduce cardiac fibrosis after MI^34^. In our screen, miR-16-5p and the members of the miR-148/152 family had the most outspoken anti-proliferative effect in cardiac fibroblasts. In line, family members of the miR-148/152 family were described as tumour suppressors^35^, while miR-16-5p inhibited the proliferation of 3T3 fibroblasts^36^. Various miRNAs caused an increase in COL1α1 area, however this was accompanied by a significant reduction in number of nuclei for several miRNAs (Supplementary Fig. S2B), confounding interpretation of their effect on fibrosis. Various miRNA mimics increased collagen area without significantly decreasing number of nuclei (including miR-146b-5p), marking the pro-fibrotic function of these miRNAs. In line, miR-146b-5p was differentially expressed in atrial fibrillation and increased collagen content in cardiac fibroblasts^37^. While having no significant effect under basal conditions, transfection of miRNA mimics for miR-148a-3p, miR-151a-3p, miR-34a-3p, and miR-511-3p reduced collagen area only upon TGFβ stimulation, indicative of a mechanism of action directed at the fibrotic response pathway. These results indicate that various miRNAs associated with the pathogenesis of heart failure, have potent effects on cardiac collagen production.

Screening for miRNA-mediated regulation of macrophage polarization, indicated by macrophage morphology^20^, identified 76 miRNAs promoting cell roundness, a hallmark of M1 polarization (including miR-146a and miR-155-5p^9,22^), while only few miRNAs decreased cell roundness (including miR-488-5p), reflecting anti-inflammatory M2-polarization. Several miRNAs affected nuclear NFκB translocation –a marker of pro-inflammatory activation^38^– including miR-30c-2-3p, miR-146b-5p, miR-210-3p, and miR-590-5p. While most of these are newly identified, the known involvement of miR-146b-5p in NFκB activation^39^ validates our results. Additionally, the miRNA-induced alterations in nuclear NFκB translocation and macrophage roundness correlated significantly, indicating that macrophage activation and polarization are linked. Several miRNAs only prevent inflammatory stimulus-induced macrophage polarisation, while having no effect under unstimulated conditions. MiR-143-3p diminished the IFNγ-induced increase in macrophage roundness, indicative of a M1 polarization-inhibiting function. In line, M1 polarized macrophages display a decreased miR-143-3p expression in comparison with M2 polarized macrophages^40^. Similarly, 16 miRNA mimics, including miR-126, increased macrophage roundness only after IL-4 stimulation. Although miR-126 has mainly been studied for its anti-inflammatory effect via the repression of VCAM-1^41^, its function as a regulator of both activators and inhibitors of the PI3K/AKT pathway is emerging, suggesting it may promote as well as attenuate inflammation, depending of the cellular context (Reviewed^42,43^). Taken together, this phenotypic screen identified several novel roles for HF-associated miRNAs in cardiomyocyte hypertrophy, fibroblast collagen production, and macrophage polarization and activation, associating them with the pathogenesis of heart failure.

Combining the results of the screens has led to identification of 17 miRNAs able to affect all three studied HF-associated processes. Interestingly, the heart-enriched miR-133b^44^, miR-199b^45^, and miR-499a^44^ are among these. MiR-210 increased cardiomyocyte cell size, fibroblast collagen area, as well as macrophage roundness suggestive of a concerted role in aggravating HF development. Interestingly, while we did not address the presence of this miRNA in all three cell types, miR-210 has been found in cardiac fibroblast-derived exosomes^14^. MiRNAs have recently emerged to be involved in intercellular communication in the heart through exosomal transfer from the host to recipient cells to regulate biological functions^13,15,46,47^. Thus, miRNAs can also act in a paracrine fashion and in this way heart-enriched miRNAs like miR-199b may expand their impact on cardiac function.

To study the mechanism underlying the regulatory function of the miRNAs with a multi-cellular function, we studied their target genes. For this, we used the publically available data deriving from the study of Spengler *et al*.^24^. This study provides a complete description of the transcriptome-wide map of miRNA targeting events in the human failing heart, comprising several benefits to predict miRNA-mRNA interaction. First, the miRNA-mRNA interaction is based on the binding of AGO2 with the transcript, and not solely on a sequence match between the miRNA seed region and the 3′UTR of target genes, providing a more biological valid interaction. Second, both miRNAs and transcripts of interest have been quantified at an abundant level in the heart, resulting in a cardiac relevant selection.

Remarkably, the identified miRNAs with a multi-cellular function share various direct target genes, suggesting a common mechanism underlying their regulating potential. We validated the targeting of the selected genes upon overexpression of miR-145-3p in cardiomyocytes, fibroblasts, and macrophages. MiRNA overexpression decreased the transcript expression of PDLIM5 and XIRP2 in various cell types, marking these genes as important players in the regulatory function of miR-145-3p. Indeed, dysregulation of PDLIM5 and XIRP2 is shown to contribute to cardiomyocyte hypertrophy and the progression of cardiac pathology^13,48^. However, not all genes were (abundantly) expressed by each cell type, and not every gene displayed a significantly down-regulated transcript expression upon overexpression. This can be explained by the fact that the study from Spengler *et al*.^24^ used the human failing heart as a source to determine the miRNA-mRNA interactome, consisting mainly out of cardiomyocytes and fibroblasts, and to lesser extent macrophages. Furthermore, miRNAs do not solely function through inhibition of mRNA stability, but can also regulate at a post-transcriptional level, affecting protein expression^49^. Therefore, we cannot exclude the involvement of additional (more abundantly expressed) target genes to explain the observed regulatory function of miR-145-3p in each given cell type.

Thirteen miRNAs with pronounced phenotypical effects that have not been functionally ascribed yet to regulate processes underlying HF were investigated for their effect on well-established gene markers to further elucidate their hypertrophy-, fibrosis-, and inflammation-regulating functions. In cardiomyocytes, observed alterations in cardiomyocyte size were in line with expression of the cytoskeletal αSKA expression. At the same time, the stress markers ANF and BNP correlated strongly with each other, increasing the insight in the hypertrophy-regulatory function of these miRNAs.

In cardiac fibroblast, transfection of miR-151a-5p mimics increased cardiac fibroblast collagen area under both basal and stimulated conditions, and stimulated CTGF expression. However, protein expression of collagen type 1 was not affected upon transfection. Strikingly, miR-145-3p mimics increased phenotypical collagen area, accompanied by increased transcript levels of α-SMA and CTGF, and increased protein expression of collagen type 1, evidencing a pro-fibrotic function. In line, miR-145-3p inhibition decreased collagen deposition in a mouse model of lung fibrosis^50^. On the other hand, miR-891a-3p decreased fibroblast collagen content, mRNA expression of CTGF, and protein expression of collagen type 1, implying the ability to inhibit the cardiac fibrotic response.

In macrophages, the accordance of alterations in cellular roundness with their inflammatory gene expression was minimal upon miRNA transfection in contrast to stimulation with IL-4, IFNγ, and LPS. The M1/M2 classification is mainly based on *in vitro* treatment with traditional inflammatory stimuli^51^ in absence of the *in vivo* chronic and multicellular environment and, interestingly, novel phenotypes of activated macrophages have been identified that do not fit this traditional classification^52–54^. Current knowledge of the complex process of macrophage polarisation may not be adequate enough to characterise observed macrophage molecular profiles, resulting in an oversimplified classification system. Both mimics for miR-486-3p and miR-223 increased macrophage roundness, induced a pro-inflammatory expression profile at transcript level, and altered the production/secretion of different cytokines, implying induction of M1 polarization and stimulation of the pro-inflammatory response. In line, increased miR-486 levels are found in sera of acute myocardial infarction patients^55,56^, vulnerable coronary artery disease patients^57,58^, and in human leukocytes deriving from sepsis patients^59^, pointing towards an immune-modulating function. Similarly, circulating miR-223 levels are increased in acute coronary syndrome patients^58^ and may predict cardiovascular death in symptomatic coronary artery disease patients^60^. MiR-223-3p is shown to be an important regulator of the inflammatory response by targeting of Mef2c^61–64^ and IKKα^65^. On the contrary, transfection of miR-488-5p into macrophages decreased macrophage roundness and inhibited transcriptional expression of inflammatory markers and almost all cytokines detectable in the supernatant. These findings imply a role for miR-488-5p in macrophage de-activation and induction of a more quiescent profile.

We conclude that our multi-cell type high-throughput screening approach of unstimulated and stimulated primary cells, allowed identification of cardiac inflammation-related miRNAs involved in hypertrophy, fibrosis and macrophage polarization in parallel, resulting in a complete description of selected miRNA-induced effects in HF pathophysiological processes. We identified previously undescribed roles of miRNAs in hypertrophy (miR-125a-5p and miR-200c-3p), fibrosis (miR-145-3p and miR-891a-3p), and inflammation (miR-223-3p, miR-486-3p, and miR-488-5p), and attribute new biological effects to various well-known miRNAs. This study defines the ability of selected miRNAs to function as a multi-cellular regulator, modifying different cellular processes driving HF in parallel.

## Materials and Methods

### MiRNA selection

MiRNA expression profiling in patients and small animal models of inflammatory cardiac disease (patients diagnosed with viral myocarditis (VM)^9^, patients which underwent cardiac transplantation (HTX)^16^, and three cardiac disease animal models: Coxsackievirus B3 induced-VM^9^, mouse HTX^16^, and the ZSF1 rat model of diastolic heart failure) was the basis for the selection of 194 differentially expressed miRNAs. Supplementary Table S1 shows all miRNAs selected for *in vitro* screening based on a significantly altered expression in one or more cardiac disease models, indicating a possible regulatory role in cardiac disease progression and development.

### Isolation and culture of primary cells

All animal experiments were approved by the ethical committee of Maastricht University (Maastricht, Netherlands) and performed according to the Dutch legislation of laboratory animals. Rat neonatal ventricular cardiomyocytes (nRCMs) and rat neonatal cardiac fibroblasts (nRCFs) were isolated by enzymatic dissociation from the hearts of 1–3 day old Wistar rats and cultured as described previously^66^. Briefly: Hearts were collected and the ventricles were consecutively digested with a mixture of Collagenase (Sigma) and Pancreatin (Sigma) in ADS buffer (120 mM NaCl, 5 mM KCl, 0.8 mM MgSO_4_, 0.5 mM KH_2_PO_4_, 0.3 mM Na_2_HPO_4_, 20 mM HEPES, 5.6 mM Glucose, pH 7.35) at 37 °C. Subsequently, obtained cell solution was pre-plated onto 162 cm Corning Costar cell culture flasks in nRCM plating medium (DMEM #11966, 17% M199 medium, 10% HS, 5% NBCS). After 1 hour of incubation at 37 °C /5% CO2, the supernatant, containing mainly nRCMs, was collected and cells therein were counted manually. Adherent nRCFs remaining in the flask were cultured for 2 more days in nRCF medium (DMEM #22320, 10% FBS).

Bone marrow-derived macrophages (BMDMs) were generated as previously described^67^, in brief: bone marrow cells were isolated from 12 week old C57BL/6-N mice and cultured in RPMI supplemented with 15% L929 conditioned medium (containing M-CSF) in petri dishes for 7 days to generate BMDMs. Cells were lifted for plating at day 8 using cell scrapers.

### Culture, transfection and stimulation of primary cell culture

#### nRCM

nRCMs were seeded into 1% gelatin coated 96-well black, clear bottom, culture plates (Corning) accompanied with the transfection complex mix. This complex mix contained Lipofectamine 2000 transfection reagent (Invitrogen) in combination with mirVana mimics (Life-Technologies; f.c. 10 nM.), prepared according to manufacturer’s protocol. After 24 h of starvation, cells were stimulated for 72 hours with 5μM phenylephrine (PE)(Sigma #P6126) or treated with PBS as control.

#### nRCF

nRCFs were seeded into uncoated 384 wells μclear plates (Greiner #781092) Day after seeding, mirVana mimics (f.c. 20 nM) were transfected into the cells using Lipofectamine RNAiMax (Invitrogen) according to manufacturer’s protocol. After 24 h of starvation, cells were stimulated with 10 ng/ml TGFβ1 (Peprotech) or vehicle (PBS) for 72 hours.

#### BMDM

Subsequent to BMDM differentiation, cells were seeded into uncoated 384 wells μclear plates (Greiner). Transfection of mirVana mimics or miRCURY LNA Power inhibitors (Exiqon) was performed using Viromer Green (Lypocalyx) according manufacturer’s protocol to give a final concentration of 20 nM. 24 h after transfection, cells were stimulated for 24 hours with 20 ng/ml IL-4 (Peprotech), 20 ng/ml IFNγ (Peprotech), 50 ng/ml LPS (Sigma) or vehicle (PBS).

### Immunostaining and Microscopy

All cell types were fixed with 4% paraformaldehyde (45 min) and treated with blocking buffer for 45 min. Only fixed nRCMs were treated with permeabilisation buffer (3% Triton X-100) for 10 minutes preceding blocking procedure. After blocking, nRCMs were incubated for 3 hours at 37 °C with the primary antibodies mouse anti-α-Actinin (1:1000, Sigma), and rat anti-α-Tubulin (1:1500, Serotec). NRCFs were treated with rabbit anti-Collagen 1α1 (1:500, Abcam) and rat anti-α-Tubulin (1:750) antibody overnight at 4 °C. BMDMs were treated with rabbit NFκB p65 antibody (1:300 Santa Cruz #sc-372) and rat anti-α-tubulin (1:750) overnight at 4 °C. After primary antibody incubation, nRCMs and nRCFs were incubated with secondary antibodies goat anti-rat AlexaFluor555 (1:750, Life Technologies), goat anti-mouse AlexaFluor488 (1:500, Life Technologies) and Hoechst (1:6000, Invitrogen) for 1 hour at room temperature. BMDMs were incubated with secondary antibodies goat anti-rat AlexaFluor555 (1:500), goat anti-rabbit AlexaFluor488 (1:750, Life Technologies) and Hoechst for 1 hour at room temperature.

### Acquisition & image analysis

For the screening experiments, image acquisition was performed using an ImageXpress Micro automated high-content screening fluorescence microscope (Molecular Devices) at ×10 magnification. For the hypertrophy, fibrosis, and inflammation screen, a total of respectively 25, 16, and 9 images were acquired per wavelength per well. Image analysis was performed using the eCognition software (Definiens, platform (Munich, Germany).

### RNA isolation and RTPCR

RNA isolation was performed according RNeasy protocol (QIAGEN, Germany), followed by reverse transcription using the Qscript cDNA synthesis kit (Quanta BIO, U.S.). Real-time reverse transcriptase-polymerase chain reaction (RT-PCR or QPCR) analysis was performed using SYBR green mix (Applied Biosystems, U.S.) to describe transcript levels of different genes. The details of the sequences and thermal cycling conditions were according to the standard protocol. Data were acquired and analysed with IQ5 software (Bio-Rad, U.S.).

### Protein extraction and Western-Blotting

Cells were lysed using two times sample buffer (25 ml 0.5 M Tris-HCL, 20 ml 100% glycerol, 20 ml 20% SDS, 35 ml Aqua Dest with 1:10 β-Mercaptoethanol). For western blot analyses, protein samples were loaded on a 10% gel (4 ml Aqua Dest, 3.3 ml 30% bisacrylamide, 2.5 ml 1.5 M Tris-HCL, pH 8.8, 0.1 ml 10% SDS, 0.004 ml TEMED. SDS PAGE was performed at 120 V for approximately 120 min, after which the gel was transferred to a PVDF membrane by blotting at 200 mA for 2 h. The membranes were blocked with 5% protifar (Nutricia) for 1 h. Primary antibody was incubated overnight in %5 BSA for Collagen type 1 (Rockland, 600-401-103). Secondary antibodies conjugated with horseradish peroxidase (HRP) against rabbit (CTS, #7074 S) were next detected using enhanced chemi-luminescence, visualized with an Artemis CCD Camera, and quantified using ImageJ.

### Cytokine and chemokine quantification

R&D Systems Mouse Cytokine Array, Panel A (Catalog #ARY006, U.S.), was used to simultaneously detect the levels of 40 different cytokines and chemokines in 700 μl supernatant of cultured BMDMs following the manufacturer specifications. Signals were detected by chemi-luminescence and were visualized with an Artemis CCD Camera and subsequently quantitated with ImageJ.

### Identification of miRNA target genes

To study the mechanism underlying the regulatory function of the 17 miRNAs proven to have a multi-cellular function, we identified shared common target genes. Rather than using in silico algorithms presenting predicted miRNA target genes (many of which appear to be not functional in validation studies), we used the publically available data deriving from the study of Spengler *et al*.^65^. This study used AGO2 crosslinking immunoprecipitation coupled with high throughput sequencing (HITS-CLIP) of bound RNA interaction sites, resulting in the detection of 4000 cardiac AGO2 binding sites across more than 2200 target transcripts. Each single AGO2-interacting transcript site is matched with a complementary seed sequence of cardiac-expressed miRNAs. We identified all targeted transcripts matching the seed sequence of the 17 miRNAs with a multi-cellular function, resulting in the identification of 1290 target genes of which 15 genes can be targeted by at least 7 out of the 17 selected miRNAs (Supplementary Table S6).

### Data analysis and statistics

The effect of miRNA transfection on different read outs was determined via calculation of the log_2_ fold change of the sample mean over the mean of the negative control mimic-transfected cells within the same condition and statistically tested using an unpaired T-test. In all three screens, identification of miRNA mimic–induced phenotypical changes was based on a statistically significant (p < 0.05) sample mean log_2_ fold change of the read out over negative control and deviating more than 2x the STDEV from the negative control mean. Only hit selection for the NFκB nuclear translocation read out in the inflammation screen was based on statistically significant mean log_2_ fold change over negative control, deviating more than 1x the STDEV from the negative control mean.

See Supplementary Materials and Methods for a more elaborate and detailed description of this section.

## Supplementary information

Supplementary Materials

Supplementary Table S1

Supplementary Table S6

## Data Availability

All data generated or analysed during this study are included in this published article (and its Supplementary Information files).
