# Supplementary material for: Functional Screening Identifies MicroRNAs as Multi-Cellular Regulators of Heart Failure
Source: Sci Rep. 2019 Apr 15;9:6055. doi: 10.1038/s41598-019-41491-9 (PMC6465262; doi:10.1038/s41598-019-41491-9)
Supplement: Supplementary file 1 — Supplementary Materials [file 41598_2019_41491_MOESM1_ESM.pdf]

Functional Screening Identifies MicroRNAs as Multi-Cellular Regulators of Heart Failure

Robin Verjans [1,2 ¶], Wouter J.A. Derks [1,2 ¶], Kerstin Korn [2], Birte Sönnichsen [2], Rick E.W. van Leeuwen [1], Blanche Schroen [1], Marc van Bilsen [1,3&], Stephane Heymans [1,4,5&*]

[1] Department of Cardiology, Cardiovascular Research Institute Maastricht (CARIM), Maastricht University, 6200 MD Maastricht, Limburg, the Netherlands.

[2] former Cenix BioScience GmbH, 01307 Dresden, Saxony, Germany).

[3] Department of Physiology, Cardiovascular Research Institute Maastricht (CARIM), Maastricht University, 6200 MD Maastricht, Limburg, the Netherlands

[4] Center for Molecular and Cardiovascular Biology, Department of Cardiovascular Sciences, 3001 Leuven, Vlaams-Brabant, Belgium.

[5] Netherlands Heart Institute, 3511 EP Utrecht, Utrecht, the Netherlands.

[¶]These authors contributed equally to this work.

[&]These authors also contributed equally to this work.

* Corresponding author:

E-mail: s.heymans@maastrichtuniversity.nl (SH)

Tel: +31 (0)43 3884304 / +31 (0)43 3882950

Fax: +31 (0)43 3882952

Supplementary Materials

Supplementary Materials & Methods

MiRNA selection

MiRNA expression profiling in patients and small animal models of inflammatory cardiac disease (patients diagnosed with viral myocarditis (VM), patients which underwent cardiac transplantation (HTX), and three cardiac disease animal models: Coxsackievirus B3 induced-VM, mouse HTX, and the ZSF1 rat model of diastolic heart failure) was the basis for the selection of 194 differentially expressed inflammatory miRNAs. Dataset 1 shows all miRNAs selected for in vitro screening based on a significantly altered expression in one or more cardiac disease models, indicating a possible regulatory role in cardiac disease progression and development. Both miRNA strands were included in the screen when the miRNA profiling data did not specify which miRNA strand (-3p/-5p) was measured.

Isolation and culture of primary cells

Rat neonatal ventricular cardiomyocytes (nRCMs) and rat neonatal cardiac fibroblasts (nRCFs) were isolated by enzymatic dissociation from the hearts of 1-3 day old Wistar rats and cultured as described previously [17]. Briefly: After decapitation, neonatal rat pups were dissected using a cut through the sternum and hearts were collected under semi-sterile conditions. Subsequently, atria were removed and the ventricles were divided into 8-12 equal parts. Ventricles were consecutively digested with a mixture of 0.3 mg/ml Collagenase (Sigma #C2674) and 0.3 mg/ml Pancreatin (Sigma # P3292) in ADS buffer (120 mM NaCl, 5 mM KCl, 0.8 mM $MgSO_4$, 0.5 mM KH_2PO_4, 0.3 mM Na_2HPO_4, 20 mM HEPES, 5.6 mM Glucose, pH 7.35) at 37°C. Every 20 min the supernatant was collected, suspended in 10% NBCS, and 10 ml fresh enzyme solution was added to the residual tissue. After 5 rounds of incubation, cell solutions were pooled, centrifuged, and re-suspended in nRCM plating medium (DMEM #11966, 17% M199 medium, 10% HS, 5% NBCS) and pre-plated onto 162cm Corning Costar cell culture flasks (Sigma #CLS3151). After incubation in a humidified 37 °C /5% CO2 incubator for 1 h, the supernatant, containing mainly nRCMs, was collected and cells therein were

counted manually. Adherent nRCFs remaining in the flask were cultured for 2 more days in nRCF medium (DMEM #22320, 10% FBS).

Bone marrow-derived macrophages (BMDMs) were generated as previously described [18], in brief: Bone marrow cells were isolated from 12 week old C57BL/6-N mice and cultured in RPMI supplemented with 15% L929 conditioned medium (containing M-CSF) in petri dishes for 7 days to generate BMDMs. Cells were lifted for plating at day 8 using cell scrapers.

Culture, transfection, and stimulation of primary cell culture.

nRCM: nRCMs were seeded into 1% gelatin coated 96-well black, clear bottom, culture plates (Corning #3603) at a density of 40,000 cells/well in 100μl seeding medium. Prior to cell seeding, 0.171μl Lipofectamine 2000 (Invitrogen #11668-019) transfection reagent (diluted 700x in OPTIMEM) was added to the wells in combination with mirVana mimics (Life-Technologies) according to manufacturer's protocol to end up with a final concentration of 10nM. The day after isolation, cells were washed and medium was replaced for 100μl serum-free experimental medium (DMEM 11966, 20% M199 medium) to starve cells for 24h hours. After starvation, cells were stimulated for 72 hours with 5μM phenylephrine (PE) (Sigma #P6126) or treated with PBS as control.

nRCF: nRCFs were lifted using trypsin/EDTA 48 hours after isolation, washed, centrifuged and seeded into uncoated 384 wells μclear plates (Greiner #781092) in 30 μl RCF medium containing 10% FBS. A seeding density of 2000 cells/well was used. After 24h and prior to transfection, cells were washed extensively using an automated washer dispenser (EL406, BioTek) and 30 μl low serum (0.1% FBS) nRCF medium was added. Lipofectamine RNAiMax (Invitrogen) was mixed with OPTIMEM and mirVana mimics (Life-Technologies) according to manufacturer's protocol and 5 μl of the mix was added to the wells, to give a final concentration of 20 nM for mimics and a dilution factor of RNAiMAX of 700x. After 24h of starvation, cells were stimulated with 10 ng/ml TGFβ1 (Peprotech hum. Recombinant TGFβ1 # 100-21) or vehicle (PBS).

BMDM: BMDMs were lifted using a scraper after 8 days of differentiation. Subsequently, cells were counted and seeded at a density of 2000 cells/well into uncoated 384 wells μclear plates

(Greiner #781092) in 30 μl BDMD medium. After 24h, medium was refreshed prior to transfection. Viromer Green (Lypocalyx, Halle, Germany) was mixed with Buffer F and mirVana mimics (Life-Technologies) or miRCURY LNA Power inhibitors (Exiqon) according to manufacturer's protocol and 5 μl of the mix was added to the wells, to give a final concentration of 20 nM mimics and a dilution factor of 1800x for the transfection reagent. 24h after transfection, cells were stimulated with 20 ng/ml IL-4, 20 ng/ml IFNγ (Peprotech), 50 ng/ml LPS (Sigma) or vehicle (PBS).

In the present study we used human miRNA mimics for transfection into rat and murine primary cells. Effect of inter-species differences is limited as sequence analysis showed that 118 out of the 194 miRNAs display a 100% sequence match between human and rodent and only 9 miRNA mimics showed a mismatch in the seed sequence (Supplementary Table S7), potentially leading to differences in their actual mRNA-targets.

Immunostaining and Microscopy

All cell types were fixed with 4% paraformaldehyde (45 min) and treated with blocking buffer (15% FBS, 0.3% Triton X-100, 0.03% Saponin in PBS) for 45 min. Only fixed nRCMs were treated with permeabilisation buffer (3% Triton X-100) for 10 minutes preceding blocking procedure. After blocking, nRCMs were incubated for 3 hours at 37°C with the primary antibodies mouse anti-α-Actinin (1:1000, Sigma, #A7811), and rat anti-α-Tubulin (1:1500, Serotec #MCA77G). nRCFs were treated with rabbit anti-Collagen 1α1 (1:500, Abcam #AB34710) and rat anti-α-Tubulin (1:750) antibody overnight at 4ºC. BMDMs were treated with rabbit NFκB p65 antibody (1:300 Santa Cruz #sc-372) and rat anti-α-tubulin (1:750) overnight at 4ºC. After primary antibody incubation, nRCMs and nRCFs were incubated with secondary antibodies goat anti-rat AlexaFluor555 (1:750, Life Technologies, #A11006), goat anti-mouse AlexaFluor488 (1:500, Life Technologies, #A11001) and Hoechst (1:6000, Invitrogen #H3570) for 1 hour at room temperature. BMDMs were incubated with secondary antibodies goat anti-rat AlexaFluor555 (1:500), goat anti-rabbit AlexaFluor488 (1:750, Life Technologies, #A11008) and Hoechst for 1 hour at room temperature.

Acquisition & image analysis

For the screening experiment, image acquisition was performed using an ImageXpress Micro automated high-content screening fluorescence microscope (Molecular Devices) at ×10 magnification with FITC, CY3 and DAPI filter blocks. For the hypertrophy, fibrosis, and inflammation screen, a total of respectively 25, 16, and 9 images were acquired per wavelength per well.

Image analysis was performed using the eCognition software (Definiens, platform (Munich, Germany). Number of nuclei was determined based on Hoechst staining for all three cell types. nRCM and nRCF cell size was quantified using an algorithm that recognized α-tubulin stained area. For nRCFs, collagen1α1 positive area was quantified as a measure of collagen production. For BMDMs, cell roundness was determined as a percentage of cells detected with a length–width ratio larger then threshold. NFκB nuclear translocation was based on p65 positive area in nucleus (Hoechst) and cytoplasm (α-tubulin stained area) and expressed as ratio of nuclear/cytoplasmic p65 staining.

RNA isolation and gene expression analysis

RNA isolation was performed according RNeasy protocol (QIAGEN, Germany), followed by reverse transcription using the Qscript cDNA synthesis kit (Quanta BIO, U.S.). Real-time reverse transcriptase-polymerase chain reaction (RT-PCR or QPCR) analysis was performed using SYBR green mix (Applied Biosystems, U.S.) to determine transcript levels of the genes listed below (Supplementary Table S8). The details of the sequences and thermal cycling conditions were according to the standard protocol. Data were acquired and analysed with IQ5 software (Bio-Rad, U.S.). The ΔΔCt method was used to analyse obtained Ct Values and make the mRNA levels relative to the appropriate control groups and corrected for housekeeping gene.

Protein extraction and Western-Blotting

Cells were lysed using two times sample buffer (25 ml 0.5 M Tris-HCL, 20 ml 100% glycerol, 20 ml 20% SDS, 35 ml Aqua Dest with 1:10 β-Mercaptoethanol). For western blot analyses, protein samples were loaded on a 10% gel (4 ml Aqua Dest, 3.3 ml 30% bisacrylamide, 2.5 ml 1.5 M Tris-HCL, pH 8.8, 0.1 ml 10% SDS, 0.004 ml TEMED. SDS PAGE was performed at 120 V for approximately 120 min, after which the gel was transferred to a PVDF membrane by blotting at 200 mA for 2 h. The membranes were blocked with 5% protifar (Nutricia) for 1 h. Primary antibody was incubated overnight in %5 BSA for Collagen type 1 (Rockland, 600-401-103). Secondary antibodies conjugated with horseradish peroxidase (HRP) against rabbit (CTS, #7074S) were next detected using enhanced chemi-luminescence, visualized with an Artemis CCD Camera, and quantified using ImageJ. Full length westernblots are displayed in Supplementary Figure S4.

Cytokine and chemokine quantification

R&D Systems Mouse Cytokine Array, Panel A (Catalog #ARY006, U.S.), was used to simultaneously detect the levels of 40 different cytokines and chemokines in 700 µl supernatant of cultured BMDMs following the manufacturer specifications. Signals were detected by chemi-luminescence and were visualized with an Artemis CCD Camera and subsequently quantitated with ImageJ.

Identification of miRNA target genes

To study the mechanism underlying the regulatory function of the 17 miRNAs proven to have a multi-cellular function, we identified shared common target genes. Rather than using in silico algorithms presenting predicted miRNA target genes (many of which appear to be not functional in validation studies), we used the publically available data deriving from the study of Spengler et al.[65]. This study used AGO2 crosslinking immunoprecipitation coupled with high throughput sequencing (HITS-CLIP) of bound RNA interaction sites, resulting in the detection of 4000 cardiac AGO2 binding sites across more than 2200 target transcripts. Each single AGO2-interacting transcript site is matched with a complementary seed sequence of cardiac-expressed miRNAs. We identified all targeted transcripts

matching the seed sequence of the 17 miRNAs with a multi-cellular function, resulting in the identification of 1290 target genes of which 15 genes can be targeted by at least 7 out of the 17 selected miRNAs (Supplementary Table S6).

Data analysis and statistics

For all analysed miRNA samples, mean and standard deviation of individual wells over 3 replicate plates was determined. Inter-plate variability was corrected by scaling each data point to the corresponding negative control mean (negative control mimic transfected cells). The effect of miRNA transfection on different read outs was determined via calculation of the $\log_2$ fold change of the sample mean over the mean of the negative control mimic-transfected cells within the same condition and statistically tested using an unpaired T-test.

In all three screens, identification of miRNA mimic–induced phenotypical changes was based on a statistically significant ($p<0.05$) sample mean $\log_2$ fold change of the main read out over negative control and deviating more than 2x the STDEV from the negative control mean. The main readouts per screen were cell size for the hypertrophy screen, number of nuclei and collagen area for the fibrosis screen, and cell roundness and NFκB nuclear translocation for the inflammation screen. Only hit selection for the NFκB nuclear translocation read out in the inflammation screen was based on statistically significant mean $\log_2$ fold change over negative control, deviating more than 1x the STDEV from the negative control mean.

Supplementary Figures

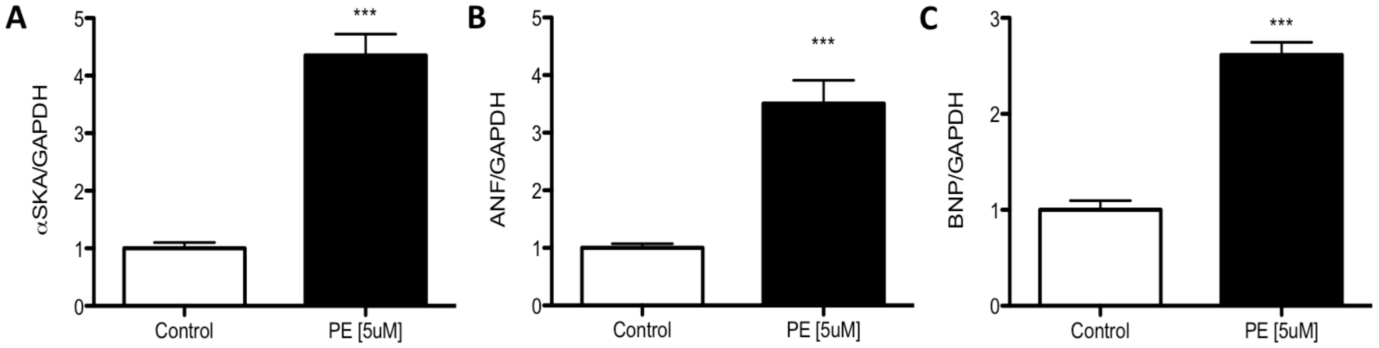

Supplementary Figure S1. Hypertrophy screening using primary neonatal rat cardiac myocytes
*Stimulation of nRCMs with PE resulted in upregulation of hypertrophic markers αSKA (A), ANF (B) and BNP (C). Data information: Values in A-C are expressed as means ± standard error, level of significance was determined using Unpaired t-test, n = 9 replicates deriving from 3 different cardiomyocyte isolation experiments, *** denotes P<0.001 versus unstimulated negative control.*

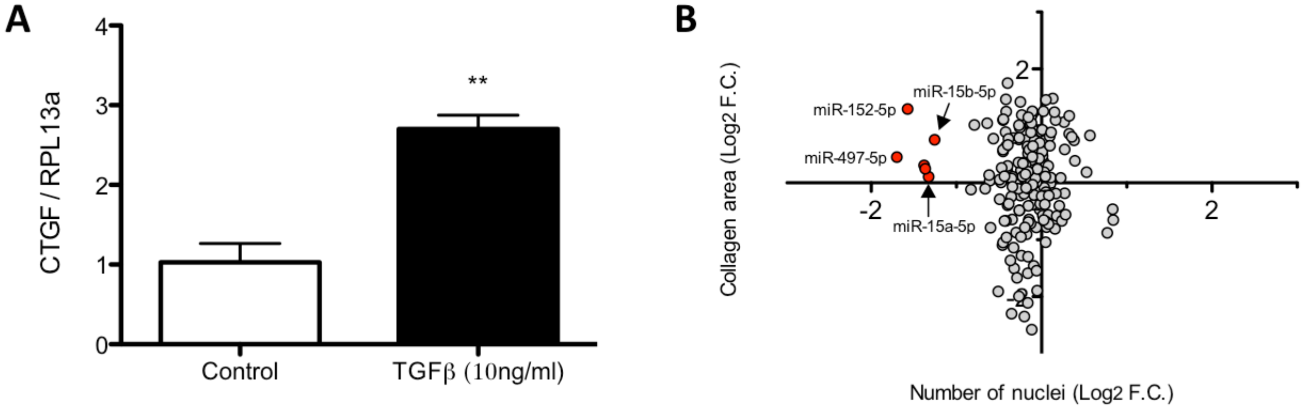

Supplementary Figure S2. Fibrosis screening using primary neonatal rat cardiac fibroblasts

*A) Stimulation of nRCFs with TGFβ resulted in upregulation of CTGF transcript levels. B) Several miRNAs gave rise to a substantial reduction in number of nuclei and, suggesting that these miRNAs promote apoptosis, complicating interpretation of their collagen increasing effect. Data information: Values in A-B are expressed as means ± standard error, level of significance was determined using Unpaired t-test, ** denotes P<0.01, n = 9 replicates deriving from 3 different cardiac fibroblast isolation experiments.*

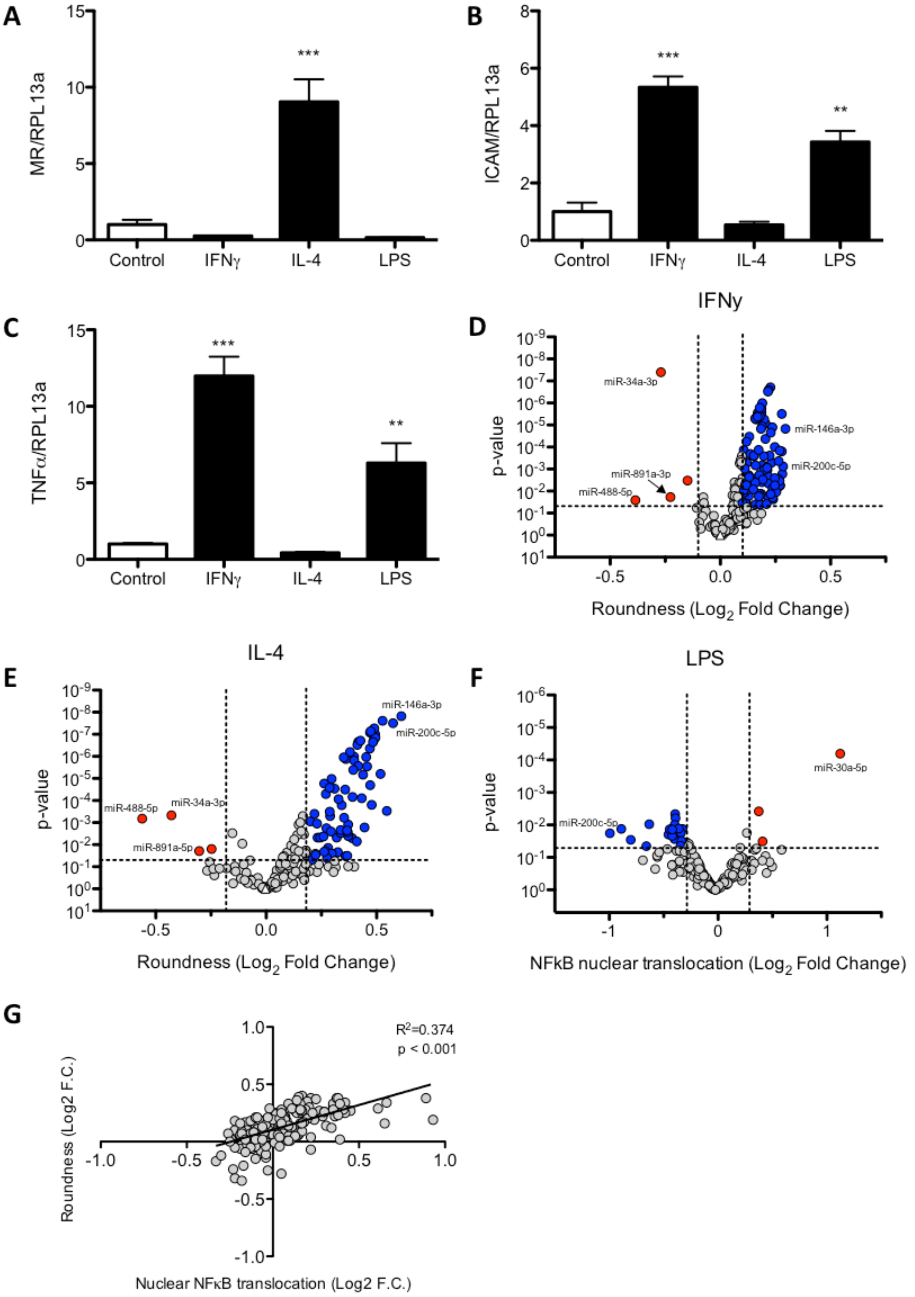

Supplementary Figure S3. Inflammatory screening using primary bone marrow-derived macrophages *RT-PCR analysis shows that IL-4 treatment increased MR (A) mRNA expression, while pro-inflammatory stimulus IFNγ and LPS both induced ICAM (B) and TNFα (C) mRNA expression. Vulcano plots showing log_2 fold change in cell roundness in macrophages polarised towards D) M1 (IFNγ) and E) M2 (IL-4). F) NFκB nuclear translocation in LPS-stimulated conditions compared to negative control mimic transfected BMDMs. Values represent log_2 fold change, normalised to negative control mimic-transfected cells within the same condition, n = 3 replicates. MiRNA mimics that significantly increased or reduced these readouts according to previously described criteria are highlighted in red or blue, respectively. G) Induced changes upon miRNA mimic transfection in macrophage roundness and nuclear NFκB translocation correlate significantly (P>0.001). Data information: Values in A-C are expressed as means ± standard error, n = 3 replicates, level of significance was determined using Unpaired t-test (D-F) and One-Way ANOVA, Post-Hoc Dunnett's Multiple Comparison Test (A-C), *** denotes P<0.001.*

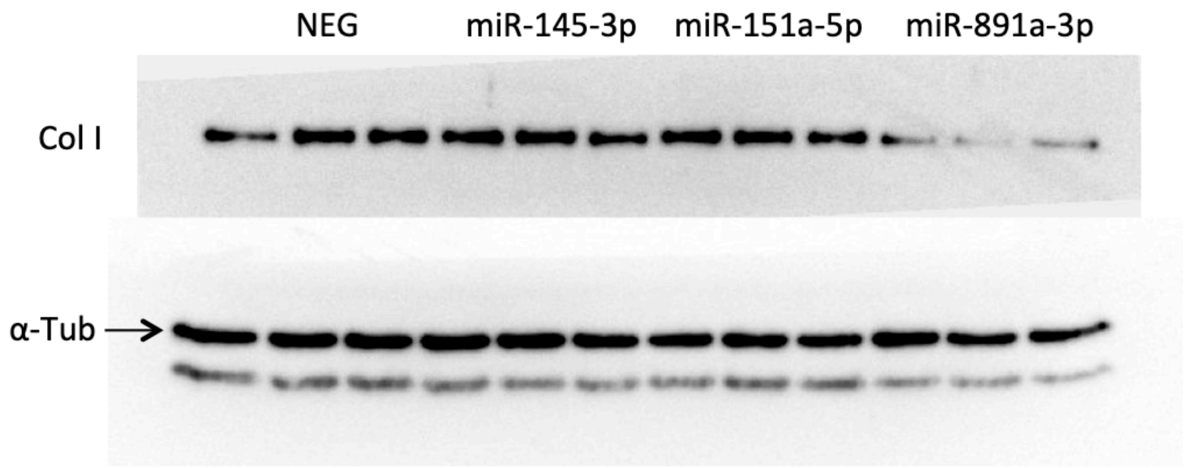

Supplementary Figure S4. ***Representative full-length western blots of collagen type I (Col I) and normalizer α-Tubulin (α-Tub) performed with cell lysates of nRCFs transfected with negative control (NEG), miR-145-3p, miR-151a-5p, and miR-891a-3p mimics****. Samples were run on the same gel and blot, different regions of the blot were cropped to quantify Col I and a-Tub, and every sample per protein was acquired under the same exposure conditions.*

Supplementary Tables

Supplementary Table S2. MiRNAs decreasing cardiomyocyte size upon PE-stimulation only.

miRNA	Unstimulated		PE-Stimulated	
	Cardiomyocyte cell size (Log2 Fc)	p-value	Cardiomyocyte cell size (Log2 Fc)	p-value
miR-130b-3p	-0.290	0.088	-0.551	0.000
miR-151a-3p	-0.148	0.263	-0.385	0.016
miR-15a-5p	-0.224	0.190	-0.488	0.002
miR-16-5p	-0.461	0.077	-0.549	0.003
miR-181a-5p	-0.223	0.198	-0.549	0.002
miR-181c-5p	-0.227	0.159	-0.489	0.015
miR-185-5p	0.000	0.970	-0.448	0.008
miR-195-5p	-0.162	0.221	-0.460	0.033
miR-200c-5p	-0.291	0.126	-0.425	0.001
miR-22-5p	-0.201	0.066	-0.402	0.014
miR-26a-5p	0.012	0.902	-0.404	0.001
miR-423-5p	-0.138	0.321	-0.424	0.000
miR-96-5p	-0.444	0.094	-0.470	0.025

Supplementary Table S3. MiRNAs increasing macrophage roundness upon IL-4-stimulation.

miRNA	Unstimulated		IL-4	
	Macrophage roundness (Log2 Fc)	p-value	Macrophage roundness (Log2 Fc)	p-value
let-7b-5p	0.160	0.093	0.263	0.021
miR-126-3p	0.024	0.738	0.288	0.000
miR-130b-3p	0.188	0.083	0.310	0.000
miR-135b-5p	0.136	0.121	0.287	0.000
miR-148a-3p	0.187	0.077	0.324	0.022
miR-16-2-3p	0.176	0.112	0.270	0.000
miR-16-5p	0.261	0.089	0.367	0.032
miR-192-5p	0.123	0.323	0.273	0.002
miR-221-5p	0.231	0.072	0.380	0.000
miR-27b-3p	0.039	0.608	0.233	0.005
miR-29b-3p	0.211	0.089	0.484	0.000
miR-29c-3p	0.098	0.345	0.338	0.003
miR-335-5p	0.247	0.070	0.370	0.003
miR-338-3p	0.158	0.200	0.391	0.000
miR-511-3p	0.083	0.144	0.209	0.047
miR-96-5p	0.120	0.108	0.353	0.001

Supplementary Table S4. MiRNAs affecting all three studied HF-associated processes.

miRNA	Cardiomyocyte Cell size (Log_2 f.c.)	Fibroblast Collagen area (Log_2 f.c.)	Macrophage Roundness (Log_2 f.c.)
let-7c-3p	-0.253	-1.069	0.338
miR-101-3p	-0.250	0.224	0.254
miR-130a-3p	-0.401	0.156	0.217
miR-133b	-0.403	0.819	0.191
miR-145-3p	-0.392	1.025	0.283
miR-152-5p	0.235	3.206	0.291
miR-15b-5p	-0.308	1.166	0.216
miR-199b-5p	-0.235	-1.310	0.239
miR-200c-3p	0.437	0.940	0.375
miR-210-3p	0.285	0.646	0.273
miR-223-3p	-0.245	-0.103	0.278
miR-29a-3p	0.195	-2.238	0.085
miR-361-5p	-0.452	-0.300	0.166
miR-486-3p	-0.540	0.154	0.207
miR-499a-5p	-0.175	-0.651	0.363
miR-891a-3p	-0.482	-1.361	-0.281
miR-9-5p	-0.404	1.406	0.250

Supplementary Table S5. MiRNAs selected for in-depth analysis induce pronounced phenotypical effects

miRNA	Hypertrophy Screen<br>Cell size<br>Mean (Log_2 f.c.)	p-value	Fibrosis Screen<br>Collagen area<br>Mean (Log_2 f.c.)	p-value	Inflammation Screen<br>Roundness<br>Mean (Log_2 f.c.)	p-value
miR-125a-5p	0.374	0.001	0.107	0.749	-0.337	0.000
miR-145-3p	-0.392	0.002	0.765	0.008	-0.266	0.002
miR-146b-5p	-0.052	0.716	1.175	0.005	0.324	0.000
miR-148b-3p	-0.162	0.050	-0.876	0.022	0.772	0.000
miR-151a-3p	-0.148	0.263	-1.137	0.066	-0.329	0.000
miR-151a-5p	-0.138	0.172	1.232	0.036	-0.097	0.023
miR-200c-3p	0.437	0.002	0.751	0.035	-0.185	0.000
miR-223-3p	-0.245	0.001	-0.807	0.001	0.158	0.000
miR-30c-2-3p	-0.127	0.550	1.015	0.001	-0.265	0.007
miR-486-3p	-0.540	0.001	-0.484	0.020	-0.262	0.001
miR-488-5p	0.099	0.291	-0.956	0.000	-0.410	0.001
miR-590-5p	0.167	0.000	0.150	0.372	-0.188	0.001
miR-891a-3p	-0.482	0.001	-1.907	0.007	-0.510	0.000

Supplementary Table S7. MiRNAs lacking a conserved seed sequence between human and rat.

miRNA	Human sequence	Rat sequence
hsa-miR-222-5p	CUCAGUAGCCAGUGUAGAUCCU	GGCUCAGUAGCCAGUGUAGAU
hsa-miR-450b-3p	UUGGGAUCAUUUUGCAUCCAUA	AUUGGGGACGCUUCGCAUUCA
hsa-miR-30b-3p	CUGGGAGGUGGAUGUUUACUUC	CUGGGAUGUGGAUGUUUACGUC
hsa-miR-345-5p	GCUGACUCCUAGUCCAGGGCUC	UGCUGACCCCUAGUCCAGUGC
hsa-miR-146b-3p	UGCCCUGUGGACUCAGUUCUGG	CCUAGGGACUCAGUUCUGGUG
hsa-miR-214-5p	UGCCUGUCUACACUUGCUGUGC	AGAGUUGUCAUGUGUCU
hsa-miR-210-5p	AGCCCCUGCCCACCGCACACUG	AGCCACUGCCCACAGCACACUG
hsa-miR-511-5p	GUGUCUUUUGCUCUGCAGUCA	CAUGCCUUUUGCUCUGCACUC
hsa-miR-135a-3p	UAUAGGGAUUGGAGCCGUGGCG	UGUAGGGAUGGAAGCCAUGAAA

Seed sequence is shown in bold and underlined. Mismatches between human and rat are displayed in red.

Supplementary Table S8. RT-PCR primer sequences

Gene	Forward sequence	Reverse sequence
Rno-BNP	GCTGCTTTGGGCAGAAGATAGA	GCCAGGAGGTCTTCCTAAAACA
Rno-ANF	ATCACCAAGGGCTTCTTCCT	TGTTGGACACCGCACTGTAT
Rno-αSKA	TCGCTGACCGCATGCA	CCGCCGATCCACACTGA
Rno-CTGF	CACAGAGTGGAGCGCCTGTTC	GATGCACTTTTTGCCCTTCTTAATG
Rno-COL1a1	CTTCACCTACAGCACCCTTGTG	CTTGGTGGTTTTGTATTCGATGAC
Rno-αSMA	GTCCCAGACACCAGGGAGTGA	TCGGATACTTCAGGGTCAGGA
Rno-GAPDH	GGTGGACCTCATGGCCTACA	CTCTCTTGCTCTCAGTATCCTTGCT
Rno-RPL13a	CTGCTGGGCCGAAAGGTGGT	GGGGCTCGGAAGTGGTAGGG
Mmu-MR	TGCCAAAAATTATTGATCCTGTAACT	CGCCGGCACCTATCACA
Mmu-ICAM	TGGAGACGCAGAGGACCTTA	CGCTCAGAAGAACCACCTTC
Mmu-TNFα	CATCTTCTCAAAATTCGAGTGACAA	TGGGAGTAGACAAGGTACAACCC
Mmu-INOS	TCAGCCACCTTGGTGAAGGGA	TCCAACGTTCTCCGTTCTCTTGC
Mmu-ARG	CAAGACAGGGCTCCTTTCAG	GCTTATGGTTACCCTCCCGT
Mmu-RPL13a	GAGCCCCCAGCCGCATTTTC	GGGACCACCATCCGCTTTTTCTTG
